# The role of surface chemistry on CO_2_ adsorption in biomass-derived porous carbons by experimental results and molecular dynamics simulations

**DOI:** 10.1038/s41598-022-12596-5

**Published:** 2022-05-26

**Authors:** Mobin Safarzadeh Khosrowshahi, Mohammad Ali Abdol, Hossein Mashhadimoslem, Elnaz Khakpour, Hosein Banna Motejadded Emrooz, Sadegh Sadeghzadeh, Ahad Ghaemi

**Affiliations:** 1 Nanotechnology Department, School of Advanced Technologies, Iran University of Science and Technology (IUST), Narmak, Tehran, 16846 Iran; 2 School of Chemical, Petroleum and Gas Engineering, Iran University of Science and Technology (IUST), Narmak, Tehran, 16846 Iran

**Keywords:** Environmental sciences, Materials science, Nanoscience and technology

## Abstract

Biomass-derived porous carbons have been considered one of the most effective adsorbents for CO_2_ capture, due to their porous structure and high specific surface area. In this study, we successfully synthesized porous carbon from celery biomass and examined the effect of external adsorption parameters including time, temperature, and pressure on CO_2_ uptake in experimental and molecular dynamics (MD) simulations. Furthermore, the influence of carbon’s surface chemistry (carboxyl and hydroxyl functionalities) and nitrogen type on CO_2_ capture were investigated utilizing MD simulations. The results showed that pyridinic nitrogen has a greater tendency to adsorb CO_2_ than graphitic. It was found that the simultaneous presence of these two types of nitrogen has a greater effect on the CO_2_ sorption than the individual presence of each in the structure. It was also revealed that the addition of carboxyl groups (O=C–OH) to the carbon matrix enhances CO_2_ capture by about 10%. Additionally, by increasing the simulation time and the size of the simulation box, the average absolute relative error for simulation results of optimal structure declined to 16%, which is an acceptable value and makes the simulation process reliable to predict adsorption capacity under various conditions.

## Introduction

Carbon dioxide (CO_2_), as a byproduct of fossil fuel combustion, is the main cause of unusual climate change and global warming^1–3^. It is estimated that only fuel-based power plants will cause a 50% increase in CO_2_ emission by 2030^4^. However, due to strong demands for fossil fuels, as an essential source of energy, CO_2_ emissions cannot be avoided. Therefore, CO_2_ capture and storage have gained significant attention in recent years and extensive research has been performed to develop materials and novel approaches for efficient CO_2_ adsorption^5^. Potential strategies for representing CO_2_ adsorption under high-pressure fuel gas streams include solvent absorption, membrane separation, pressure swing adsorption (PSA), and temperature swing adsorption (TSA). PSA is a potential choice because of its simplicity and convenience of operation, low cost, energy-saving (No heating required for regeneration), and economic feasibility, which is especially beneficial in the case of medium- and small-scale activities^6–8^. PSA technology is a cyclic adsorption process in gas separation employing different adsorbents and adsorption capacity rates. The type of adsorbent is crucial in this procedure for attaining excellent separation performance^9,10^. As a result, various solid adsorbents including MOFs (metal organic frameworks), zeolites, porous polymers, functionalized porous silica, metal oxides, functionalized activated carbon, and porous carbons have been verified to be proper for this purpose^11^. Because of the outstanding textural features, high surface area, adjustable porosity, high stability, and low cost, biomass-derived porous carbons are regarded as the most desirable adsorbents for CO_2_ capture^12^.

Porous carbons are commonly employed in environmental and energy applications^1^. They have a lot of potential as catalyst supports and matrices for gas capture, storage, and separation^2,3^. Increasing the surface area, pore structure, and surface chemistry of synthetic porous carbons has recently resulted in the development of new types with improved CO_2_ adsorption capacity. CO_2_ capture can also be adjusted by applying a specific synthesis method and adding functional groups such as nitrogen, oxygen, and sulfur^13,14^. Especially, several researchers have suggested that the presence of narrow micropore volume in porous carbons enhanced their CO_2_ uptake capacity^15^.

**Figure 1:**
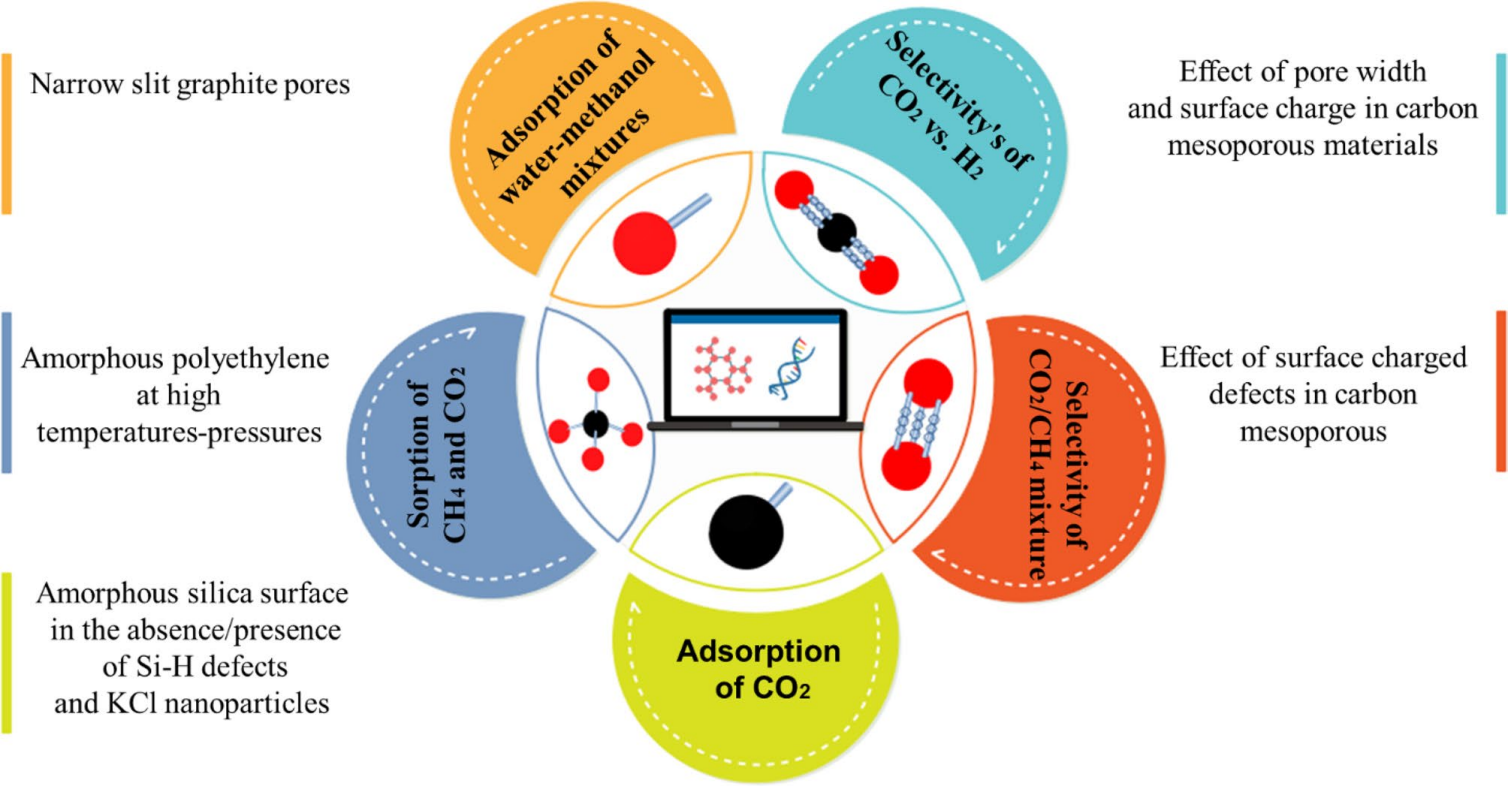
An overview of the simulation of various gases-liquids capture on solid adsorbents in previous studies^25–28^.

CO_2_ molecules are selectively adsorbed onto the surface of adsorbents in adsorption processes when no electron transfer occurs between the adsorbate and the adsorbent. The phenomenon of physisorption of gases occurs when Van der Waals forces keep molecules for far longer than they can on an open surface, making it easier to desorb CO_2_ and regenerate adsorbents for reuse^16^. Since adsorption is a complicated behavior, it is critical to be investigated different adsorbents. Furthermore, it is difficult to examine the adsorption values at non-measurable temperatures and pressures. Thus, it is required to predict them at the industrial and nanoscale. As a result, molecular simulation has been utilized as a complementary technique to the experimental measurements. It provides essential deep insight into the adsorption details and molecular interactions between different components of a system as an additional source of property data. Molecular dynamics (MD) or Monte Carlo (MC) approaches can be used to estimate gas solubility- adsorption using microscopic methodologies^17,18^. The most accurate simulation technique among the various simulation approaches is molecular dynamics, which may be ascribed to the method's degree of freedom. The approach in MC is stochastic (probabilistic), but the method in MD is deterministic. The direct motion of molecules and their collisions with walls and other molecules are taken into account in MD. In general, this approach is based on Newton's second law, and the route of the particles is calculated by integrating this equation. The macroscopic parameters of the system may be obtained by getting the particle's route, motion, and velocity, and then averaging the computed values^19^. MD simulations, including ab initio MD (AIMD), reactive MD (RxMD), and nonreactive classical MD, can generate an electronic or atomistic level insight into the structural and dynamic features for predicting gas diffusivities. This method is a stable and adaptable methodology that allows users to trace a system's entire dynamical course through space and time^20,21^. Also, grand canonical Monte Carlo (GCMC) simulation can be used to determine the saturation amount under different temperature and pressure values. The heat of adsorption can also be computed simply using the adsorption amount. Research has been carried out to determine the factors that influence the amount of CO_2_ adsorption onto various materials^22^. Microporous carbon with oxygen functional groups was produced by hydrothermally treating biomass activation, according to Xiancheng Ma et al. In this case, the GCMC simulation estimated that oxygen groups and pore structures were 63% and 37% respectively responsible for CO_2_ adsorption. It also clarified that oxygen functional groups held CO_2_ by electrostatic interactions^15^. Furthermore, Chen et al. performed the GCMC and the MD simulations to study the adsorption and diffusion behavior of CH_4_ in shale nanopores with different pore diameters over a pressure range up to 20 MPa and at a specific temperature. This model provided predictions about space distribution characteristics such as free zone and adsorption zone distributions, gas number distribution, gas density distribution, free and absorbed gas proportion^17^. Leebyn Chong et al. also used the MD and the MC simulations to investigate and compare CO_2_, CH_4_ adsorption in immature type II kerogen. CH_4_ and CO_2_ showed similar adsorption in matrix micropores due to their similar swelling ability and tight confinement environment. More uptake of CO_2_ in comparison with CH_4_ in kerogen was found to be due to the meso-sized porosities^23^. Xinran Yu et al. determined carbon nano-slit void volume using the GCMC simulations and acquired proper experimental circumstances for mitigation of helium adsorption effect. Additionally, they examined helium capture and its local density in a pore^24^. Figure 1 shows an overview of the various gases-liquid captured on solid adsorbents in previous studies^25–28^.

In this study, we combined the experimental analyses and MD simulation to study CO_2_ adsorption on the porous carbon derived from celery biomass, focusing on the underlying mechanism of physisorption. We applied MD simulation using LAMMPS software (Large-scale Atomic/Molecular Massively Parallel Simulator) to determine a randomly void based on CO_2_ molecules and investigate its adsorption. The morphology and structure have been characterized by high-resolution transmission electron microscopy, Raman, X-ray diffraction, Fourier transform infrared (FTIR), N_2_ adsorption–desorption, and X-ray photoelectron spectroscopy (XPS). In both situations (experiment and simulation), CO_2_ adsorption was investigated at temperatures of 298, 308, and 318 K, under the pressure range of 2–9 bar to assess the simulation's performance. Due to the lack of experimental data, the amount of adsorption was first investigated by increasing the temperature and pressure, and then this quantity was predicted using simulation at different ranges of temperatures and pressures. Furthermore, the impacts of different types of nitrogen including pyridinic and graphitic, surface chemistry, and size of the simulation box on the CO_2_ adsorption were investigated utilizing different structures inside the simulated condition. Eventually, using the average absolute value of relative error (AARE %), the accuracy of measurement results was verified. The MD simulation approach is applied because it can effectively predict the atomic level transport phenomenon that is supported by atom/molecule mobility.

## Materials and methodology

### Identification of porous carbon composition and structure

Porous carbon was synthesized from celery biomass wastes at 700 °C and in 3 h by a one-step self-activating method without the need for an extra reagent (Details of synthesis were described in the previous article)^29^. The final product was pickled with 1 M HCl to remove the remained impurities and then cleaned with deionized water until neutral pH. Afterward, it was dried in an oven at 90 °C and its final yield reached 13%. The synthesized sample was named C-700 where 700 was the pyrolyzation temperature. All of the chemicals-gases that were used are listed in Table 1. The collection of celery was following the relevant institutional, national, and international guidelines and legislation. Permission for the plant sample collection was obtained from the Forest Association, Tehran. Structural, textural, and chemical characteristics of the synthesized carbon have been investigated as follows: For structure determination of the synthesized powder, X-ray diffraction analysis was performed on BRUKER D8 ADVANCE diffractometer with Cu K_α_ (λ = 1.54 Å). Micromeritics ASAP2020 (US) adsorption analyzer was employed to measure the N_2_ adsorption–desorption isotherms at 77 K for the determination of specific surface area, pore volume, and pore size distribution. Before performing the adsorption–desorption analyses, the prepared sample was degassed under dynamic vacuum conditions for 6 h at 150 °C. Fourier transform infrared (FTIR) spectroscopy was conducted with KBr pallets on Perkin–Elmer Spectrometer to distinguish surface functional groups. X-ray photoelectron spectroscopy (XPS) analysis was accomplished by XPS Spectrometer Kratos AXIS Supra and using an Al Ka source to determine the types of functional groups and elemental compositions. Raman spectroscopy was conducted on a Takram micro-Raman spectrometer (Teksan™, Iran). High-resolution transmission electron micrographs were observed on a 300-kV FEI (US) TITAN microscope (HR-TEM).

**Table 1:** List of chemicals and gases used in this article.

Chemical name	Purities (%)	CAS.no	Sources
CO_2_	99.99	124-38-9	Arman gas
N_2_	99.99	7727-37-9	Arman gas
HCl	37	7647-01-0	Dr. Mojallali™
Deionized water	99.99	7732-18-5	IUST

### CO_2_ adsorption measurement

Before measuring the sorption isotherms, the sorbent was heated at 180 °C overnight under a turbo-pumped vacuum to ensure the complete removal of the pre-adsorbed CO_2_ gas and the impurities. A fixed bed adsorption reactor was employed to assess the CO_2_ adsorption–desorption performance of C-700. It records the momentary changes in temperature and pressure by connecting to a data analyzer, as shown in Fig. S1. N_2_, as a purge gas, was injected into the device compartment to remove the moisture, evacuate and degas the device, and check that no connections were leaking. A 1 g prepared sample was placed in the device cylindrical compartment and sealed completely. The experiments were carried out for 60 min at temperatures of 298, 308, and 318 K and under the pressure range of 2–10 bar. Due to the existence of a mixing tank in the passage, the CO_2_ pressure and temperature were stabilized. The stable gas was then fed into the adsorbent reactor. An electrical heat tracer also provided heat to the reactor. The real-time variations of CO_2_ temperature–pressure were regulated and recorded using the control panel and computer.

### Methodology to simulate the porous carbon

**Figure 2:**
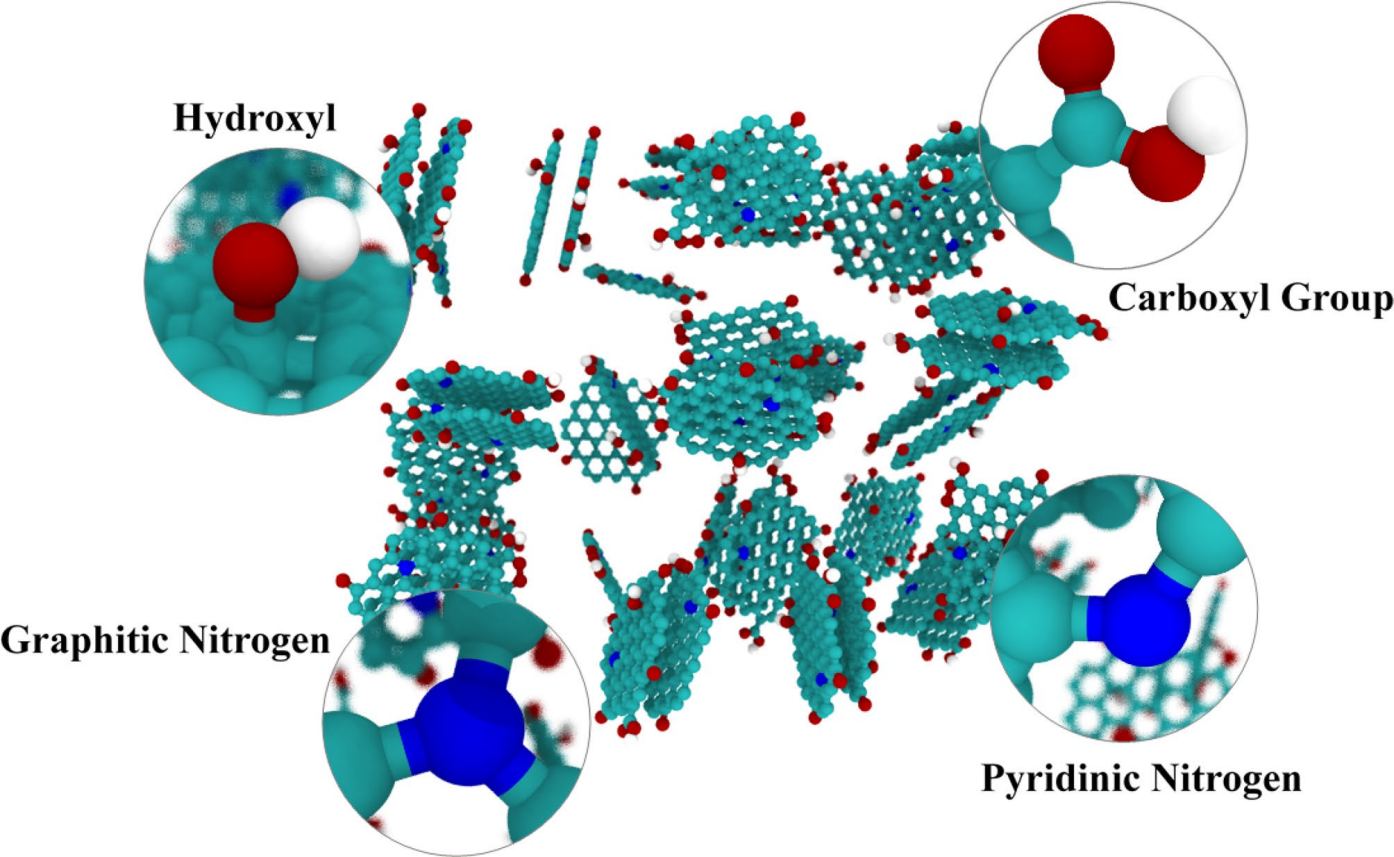
Schema of the groups involved in the molecules studied.

All the simulations were performed with the Large-scale Atomic/Molecular Massively Parallel Simulator (LAMMPS) package^30^. Visual outputs and pictures were extracted by VMD software^31^. Primarily, we used a box with 180 nm * 60 nm * 60 nm dimensions, then we increased the box size to 540 nm * 60 nm * 60 nm and 1020 nm * 60 nm * 60 nm, respectively to investigate the effect of the simulation box enlargement on the adsorption of CO_2_ molecules. It is noteworthy that the density of CO_2_ molecules was similar in all the box dimensions. The time step of the simulations was 2 fs (0.5 ns for plotting). To simulate porous carbon, a graphitic flake structure was chosen which is a common model and used in different researches^32,33^. The pore size distribution, micro-porosities, and concentration of some oxygenated groups were introduced as limitations but the constructed model preserved some key features such as pores disorder, surface chemistry, and heterogeneity of the structure. To resemble the desired surface area and density which are about 1200 m^2^ gr^−1^ and 300 kg m^−3^ respectively (The detailed information is clarified in the result and discussion section, Table 3), we put a mixture of 24 double-layer and 6 single-layer nanoflakes of graphene together using Packmol package^34^ according to our calculations. For taking into account the micropore structure of porous carbon we put individual flakes with a minimum of one nm distance. The flakes are doped with graphitic and pyridinic nitrogen and functionalized with hydroxyl and carboxyl groups in a specified ratio as displayed in Fig. 2, so that they can fairly accurately model the porous carbon specimen. This structure was treated as rigid during simulations. In this simulation, all of the interactions between different atoms were modeled using Lennard–Jones (LJ) potential, and the bonds and angles between individual molecules were molded with spring. The LJ potential is given using Eq. (1):

1 $$U_{LJ} = 4\varepsilon \left[ {\left( {\frac{\sigma }{r}} \right)^{12} - \left( {\frac{\sigma }{r}} \right)^{6} } \right]$$

The standard Lorentz-Berthelot combining rules^35–37^ were used for computing compounds of LJ parameters. The Lorentz- Berthelot combination rule is given using Eq. (2–3):

2 $$\sigma_{ij} = \frac{{\sigma_{ii} + \sigma_{jj} }}{2}$$

3 $$\varepsilon_{ij} = \sqrt {\varepsilon_{ii } \varepsilon_{jj} }$$

The corresponding parameters and the charges of the atoms and ions are listed in Table 2. The long-range coulombic interactions were computed by a particle mesh (pppm) solver. The standard coulombic interaction potential (lj/cut/coul/long) is calculated using Eq. (4):

4 $$E = \frac{{Cq_{i } q_{j} }}{\varepsilon r}\quad r < r_{c}$$

where C is an energy-conversion constant, Qi and Qj are the charges on the two atoms, and ϵ is the dielectric amount that can be set by the dielectric command. The cutoff r_c_ shortens the interaction distance. A cutoff radius of 12.0 Å was applied to the LJ interactions. Electrostatic interactions within the cut-off radius are computed directly, and outside of this distance are calculated according to the reciprocal space. The boundary conditions of the system were periodic in all directions. Before the main simulation was started, CO_2_ molecules had been relaxed at 298 K temperature under the Langevin thermostat for 50 ps. During the simulation, the graphene flakes were kept fixed. Afterward, we performed the simulation at different temperatures in the range of 253 to 373 K and under pressures varying from 1 to 10 bar to investigate the adsorption in various conditions. The process’s pressure was calculated using the Van der Waals equation (Eq. 5):

5 $$P = \frac{RT}{{V - b}} - \frac{a}{{V^{2} }}$$

where R is the universal gas constant, T is absolute temperature, a and b parameters are the gas constants, and V is the molar volume, respectively. In this specific simulation for introducing and controlling the pressure, we used the Van de Waals equation. In this equation, the exact number of CO_2_ molecules in the system is described by the different parameters of the equation including pressure, temperature, and volume of the system. In this equation pressure and the number of molecules have a linear relationship with each other, thus for reach higher pressure we need more CO_2_ molecules in the system and vice versa. The adsorption of CO_2_ gases is completely a physical process here so we don't expect any bond formation, rather the adsorption is described by the residence time of molecules and their interactions with the surface of porous carbon and functional groups. Hence we monitor the residence time of the molecules and their density in the whole simulation box and inside the porous structure in every step of the simulation to calculate adsorption. An important phase of every MD simulation is equilibration. In this stage, we simulate for a short period and let the particles of the system interact with each other so that they can find and go to their equilibrium position and minimize the total potential energy of the system. Once the potential energy of the system has stabilized, we can perform the main simulation.

**Table 2:** Lennard Jones parameters and partial charges.

Molecule/functional groups	Elements	Epsilon (ev)	Sigma (Å)	Charges (q)	References
Graphene	C	0.00372	3.3997	0.0	^38^
Graphitic	N	0.00335	3.26	− 0.94	^33,38^
	C(N)	0.00372	3.3997	0.3133	
Pyridinic	N	0.00737	3.250	− 0.6696	^38,39^
	C(N)	0.00372	3.3997	0.3348	
Hydroxyl	C	0.00304	3.55	0.2	^38^
	O	0.00672	3.07	− 0.64	
	H	0.0	0.0	0.44	
Carboxyl	C	0.00448	3.75	0.63	^40^
	O(H)	0.00737	3.0	− 0.58	
	H(O)	0.0	0.0	0.45	
	O(C)	0.00910	2.96	− 0.50	
Carbon dioxide	C	0.002424	2.757	0.7	^41^
	O	0.006938	3.033	− 0.35	

## Result and discussion

### Textural and surface chemical properties of the porous carbon

**Figure 3:**
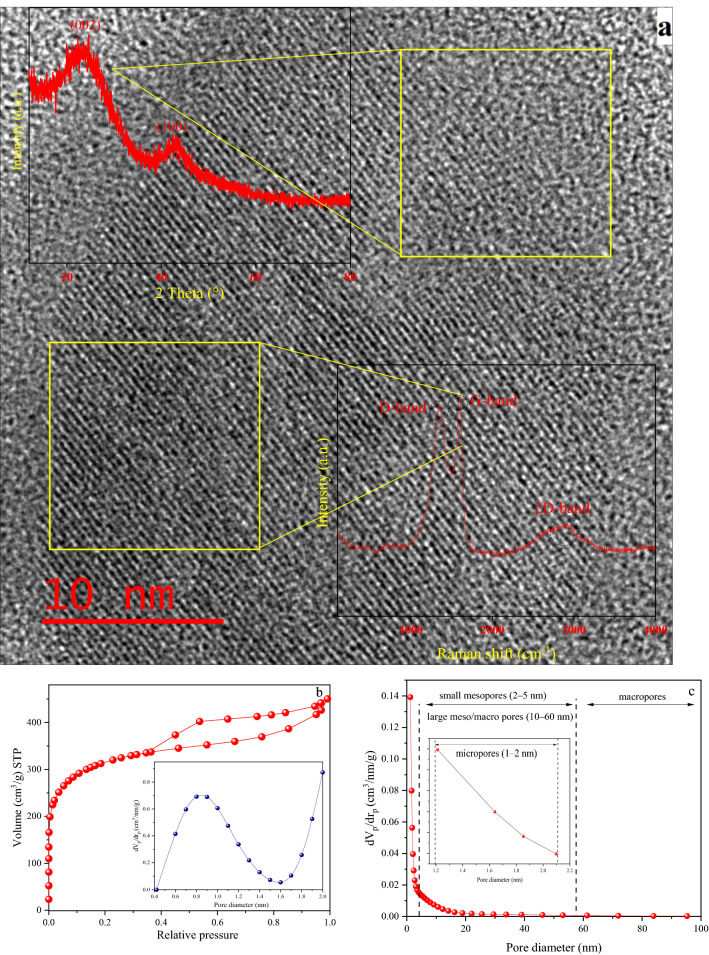

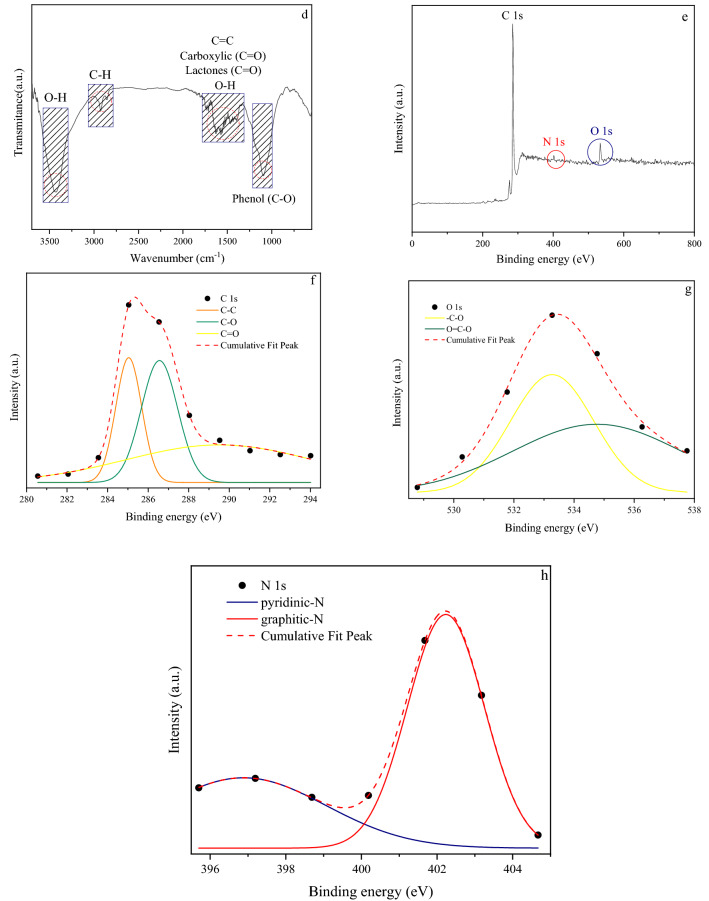
(**a**) XRD, HRTEM, Raman (**b**) N_2_ adsorption/desorption isotherms; (b-inset) MP plots (pores smaller than 2 nm); (**c**) BJH pore size distributions curves; (**d**) FTIR transmission; (**e**) XPS survey spectrum showing the presence of C, N and O elements in the structure; (**f**) C1*s*, (**g**) O1*s*, and (**h**) N1*s* peak deconvolution of C-700.

The textural characteristics, morphology, and surface chemical properties of the porous carbon (C-700) have been depicted in Fig. 3. As can be observed in Fig. 3 (a-Top-left), the XRD pattern clearly shows the development of turbostratic carbon with structural ordering intermediate between amorphous carbon and crystalline graphite. XRD of C-700 shows two broad peaks at around 22–23° and 42–44°, which belong to the graphitic carbons' (002) and (100) planes, respectively. HR-TEM micrograph (Fig. 3 (a-background)) shows an amorphous-crystalline intermediate structure which is consistent with XRD results. Moreover, D-band peaks at approximately 1345 cm^−1^ and G-band peaks at around 1603 cm^−1^, respectively, indicate the existence of a carbon lattice defect with deformed structure (sp3) and some order-layered graphite with vibration sp2 hybridized carbon atoms, according to Raman spectroscopy (Fig. 3 (a-down-right)). Furthermore, the existence of a 2D-band about 2873 (cm^−1^) indicates the presence of a graphene-like structure locally.

Figure 3b,c represent the N_2_ adsorption/desorption isotherms, micropores plot (MP), and pore size distributions (PSD) of C-700, respectively. C-700 possesses a specific surface area of 1126 m^2^ g^−1^, a mean pore diameter of 2.5 nm, and a total pore volume of 0.69 cm^3^ g^−1^. There is an IV hysteresis loop in the isotherm of C-700 in the IUPAC classification (Fig. 3b). This indicates that a large number of mesopores were formed, which might be due to the stacking of carbon layers. Nitrogen adsorption capacity increases significantly at low pressures (p/p_0_ < 0.01), followed by a lengthy peak, that suggests the presence of a large number of micropores. At p/p_0_ > 0.4, capillary condensation occurs, revealing the materials' distinctive meso-microporous characteristics^42–45^. Based on MP and BJH plot results in Fig. 3 (b-inset), and (c), C-700 has 0.27 cm^3^ g^−1^ and 0.42 cm^3^ g^−1^ mesopore and micropore volume. Based on BJH pore size distribution, porous carbon is composed of micropores (1–2 nm), small mesopores (2–5 nm), and large mesopores/macropores (10–60 nm). As a result, C-700 may be classified as hierarchical^46,47^. The Surface functional groups of samples were further conducted by FTIR analyses, as seen in Fig. 3d. It can be seen that the spectrum shows an obvious peak at ~ 3420 (cm^−1^), 2925 (cm^−1^), and 2856 (cm^−1^), which are consistent with the presence of the O–H stretching vibration in hydroxyl and carboxyl groups, asymmetric and symmetric C-H methylene, and methyl groups in aliphatic CH, CH_2_, and CH_3_ from lignin, respectively. The 1730 and 1600 (cm^−1^) bands are caused by the stretching of C=O bonds in lactone and carboxyl acid- derivatives and O–H groups, respectively. A band around 1560 cm^−1^, is corresponded to conjugated C=C and a very broad peak between 1200 and 900 cm^−1^ represents the C–O tensile vibration band in the ether, phenol, and alcohol^48–50^.

XPS analysis was held to determine the evolution of chemical states and elemental compositions on the adsorbent’s surface. The spectra of the porous carbon indicate the existence of carbon, nitrogen, and oxygen species and quantities as can be seen in the Fig. 3e–h and Table 3, respectively. The survey analysis is in the Fig. 3e and Table 3, confirm three distinct peaks located at around 285, 401.5, and 533 eV ascribing to carbon (93.7%), nitrogen (1.49%), and oxygen (4.8%), respectively. Deconvoluted spectra of C1*s* in Fig. 3f indicate that there are three peaks at 285, 287, and 289 eV, corresponding to C–C (graphitic carbon) bond, C- O (phenolic) bond, and C=O (carboxyl) bond, respectively. C-700's O1*s* spectra (see Fig. 3g) are fitted into three peaks positioned at 533 and 534.5 eV, which may be assigned to the C–O and O=C–O groups. Furthermore, there are two observable graphitic (N-Q)—Pyridinic nitrogen bonds for N1*s* (Fig. 3h)^13,51,52^.

**Table 3:** Material characteristics of simulation and experimental work.

Sample	Total BET specific surface area (m^2^ g^−1^)	Average pore diameter (nm)	Total pore volume (cm^3^ g^−1^)	Mesoporous volume (cm^3^ g^−1^)	Microporous volume (cm^3^ g^−1^)	C (%)	O (%)	N (%)
Initial simulation	1200 ± 100	1 >	NA	NA	NA	93.3	5	1.7
Optimal structure	1240 ± 115	1 >	NA	NA	NA	93.3	5	1.7
C-700	1126.2	2.4666	0.694	0.27	0.42	93.67	4.84	1.49

### Comparison between simulation and experimental data

**Figure 4:**
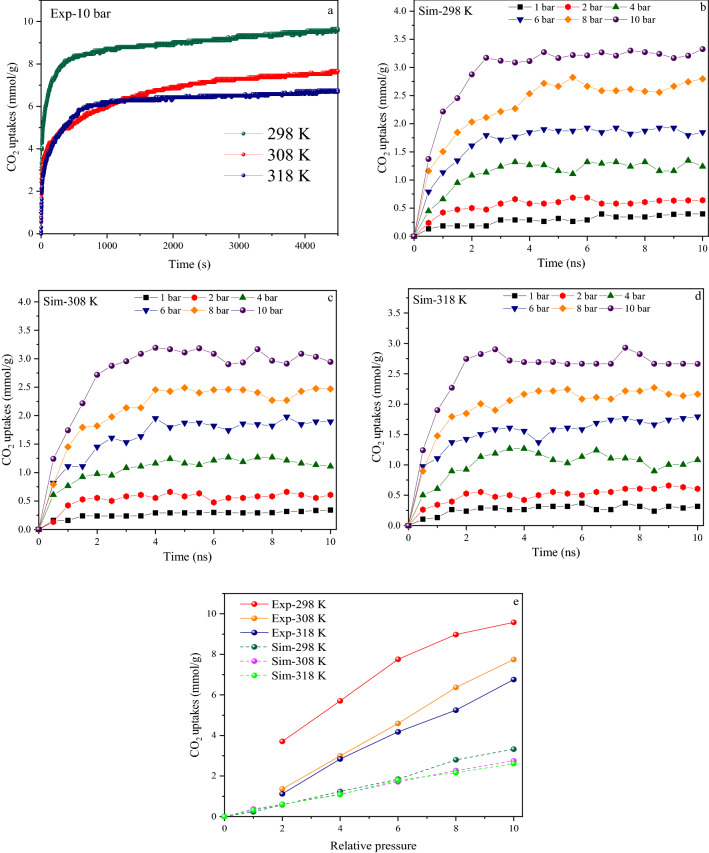
(a) Experimental CO_2_ Adsorption isotherms under 10 bar pressure at 298–318 K for 4500 Sec. (**a**); (**b**), (**c**), and (**d**) Simulation CO_2_ adsorption capacity at 298 K, 308 K, and 318 K, in the range of 1–10 bar pressure for 10 ns; (**e**) Comparison of experimental and simulation CO_2_ adsorption isotherm in the range of 1–10 bar.

The synthesized porous carbon structure contains graphitic nitrogen and hydroxyl groups, as per the results of the characterization section. Therefore, both of these were incorporated on the simulated sample's surface. In this work, each simulation was performed four times to reduce inaccuracy. One of the most important factors affecting a material's adsorption and diffusion performance is its specific surface area. Adsorbents with large open volumes and surface areas have high gas adsorption capability^32^. To compare the amount of CO_2_ adsorption in these two situations (Experimental-Simulation), the specific surface area and the percentage of identical surface elements were assumed to be the same based on the BET and XPS data. Figure 4 shows the amount of CO_2_ uptake in experimental and simulated conditions, in terms of time and pressure under different temperatures. The quadrupole nature of the CO_2_ molecule has been proposed as a beneficial property for producing a surface interaction with porous carbon via the dispersion and induction processes^12^. As can be seen from Fig. 4a, with increasing adsorption time, initially the adsorption of CO_2_ on the sorbent increases with a very steep slope and after reaching a plateau, its rate remains constant, which is kinetically consistent with the results of the simulated sample (within the simulation timeframe) (see Fig. 4b–d). At the beginning of the adsorption process, a large number of empty high-affinity adsorption sites are present on the adsorbent surface and the CO_2_ molecules are in direct contact with the adsorbent, creating strong forces between the adsorbent-adsorbate (gas–solid interactions). As the adsorption process continues, the porous carbon pores are occupied, thus causing a weaker interaction between the sorbent and the gas molecules^53,54^. The CO_2_ adsorption capacity of C-700 was measured at temperatures ranging from 298 to 318 K, indicating that lowering the adsorption temperature can improve CO_2_ adsorption capacity (see Fig. 4a). The increase in interaction is due to a rise in the kinetic energy of the gas–solid molecules involved in the adsorption process, which causes higher molecular interaction and reduces the efficient adsorption surface, according to the Boltzmann equation. As a result, the adsorption capacity gained during the adsorption process is most beneficial at the lowest temperatures, which matches the results of the simulated sample (Fig. 4b–d)^55,56^. The highest adsorption on the C-700 and simulated model is 9.57 and 3.39 (mmol g^−1^), respectively, at 10 bar pressure and 298 K. At higher temperatures, the bonds between the sorbent-adsorbate weaken and the converse repulsion becomes more favorable, resulting in a change in equilibrium in the opposite direction of the adsorption process. As the adsorption temperature rises, molecule activity increases, and the competition for reaching the restricted adsorption sites intensifies. Thus the molecules' repulsions increase and it results in the decrease of the adsorption amount. Afterward, we validated the above simulation procedure by comparing the predicted and the experimental CO_2_ adsorption data at temperatures in the range of 298–318 K and under pressures from 1 to 10 bar (Fig. 4e). The experimental results show that under the pressures from 2 to 6 bar, the adsorption values at temperatures of 308 and 318 K are almost the same by varying between 4.1 and 4.2 mmol g^−1^. But under pressures above 6 bar, the adsorption amounts show a greater increase at 308 K (6–7.2 mmol g^−1^). Similar results can be seen at 298 K in the simulated conditions. The overall amount of gas adsorption depends on both the CO_2_-structure interaction energy and the free volume (or pore space). The pore space has a major role in process of adsorption^47^. As the pressure increases, the force exerted by the pressure to move the molecules from the surface into the interconnected pores increases, and the larger space of the pores facilitates this transport. In other words, the force exerted by the adsorbing molecules on the adsorbed molecules on the surface allows more gas to be trapped by the pores. Subsequently, in the process of simulating incoming forces of the type of add-force or set-force, it results that all the pores are filled at a very high speed at first. As a result, the rapid increment of adsorption at the beginning of the adsorption process at high pressures becomes more justifiable^57^. Figure S2 shows the adsorption–desorption curves of the simulated structure at 298 K and under 10 bar as a function of simulation run time. To perform this test, we re-ran the structure that had adsorbed CO_2_, so the box got empty of the gas. The molecules which separate from the surface and enter the space of the box were deleted and the desorption capacity was calculated. It is clear that the lower gradient of desorption vs. adsorption is due to the predominance of energy between the surface and the CO_2_ molecules. The time-dependent evolution of the gas molecules was observed in three regions. The following are the outcomes: (1) CO_2_ molecules have physically adhered to the surface with the least possible energy; (2) They fill the surface; (3) And when the pores and layers become stable, they start to desorb^58^. Utilizing the trajectories recorded during the MD runs, The average residence time of CO_2_ molecules on the surface was determined to be 2 ns^59^. The predicted capture using only LJ interactions is significantly lower than the experimental outcome, according to the findings. Incorporating columbic interactions between gas molecules allows for a significantly greater agreement between simulation and experiment when estimating saturated loading^60^.

### Effect of different nitrogen type on the adsorption performance

**Figure 5:**
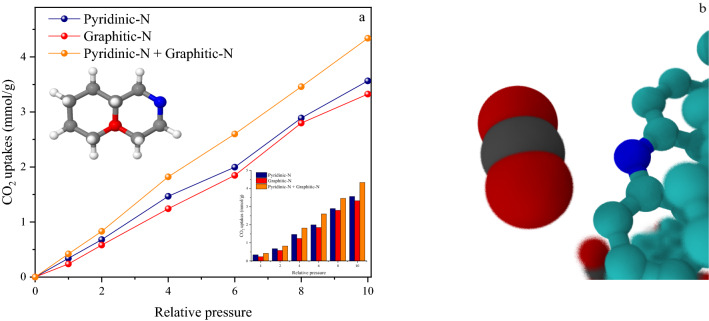
(**a**) CO_2_ uptake isotherms simulation at 298 K under pressure range up to 10 bar on generated structure within the presence of Pyridinic-N (Blue), Graphitic-N (Red) and mixed of them (Orange); (**b**) Representative snapshot of CO_2_ (Red–Black) nearby Pyridinic-N.

As proposed, the introduction of self-doped heteroatoms like nitrogen to porous carbon framework increases its surface interaction with CO_2_ molecules effectively and thus results in improved capacity and selectivity for CO_2_ capture^61,62^. It is mainly believed that the covalently tethered nitrogen functional groups act as Lewis's basic active sites for anchoring to the acidic CO_2_ molecules^63,64^. However, the adsorption mechanism is still controversial. It is also claimed that the process may be based on the interaction between highly electrostatic N-doped carbons and CO_2_ molecular quadrupole moment, which is created in the electrostatic field around the N-containing carbon surface. Another probable mechanism is the occurrence of hydrogen bonds between CO_2_ molecules and the hydrogen atoms (NH and CH) on carbon’s surface^65^. In a nitrogen-containing carbon, only a few carbon atoms of a carbon-rich matrix are substituted by nitrogen atoms, so different sorts of nitrogen functionalities on carbon’s surface are created^66^. The XPS results that were investigated in the characterization section show that the nitrogen functionalities of the C-700 porous carbon are graphitic-pyridinic. To examine the role of nitrogen via simulation in a more appropriate way, pyridinic nitrogen was substituted for graphitic type in the simulated structure to find the separate role of each nitrogen group. Simulation results indicated that pyridine nitrogen was the most effective contributor to CO_2_ capture, as illustrated in Fig. 5a. Figure 5b shows the presence of pyridinic nitrogen in the simulated structure. In addition, the simultaneous presence of these two types of nitrogen indicates that the uptake is greatly increased (4.34 mmol g^−1^). The adsorption capacity was 3.39 mmol g^−1^ in the presence of graphitic nitrogen, while in the structure with pyridinic nitrogen it reached up 3.72 mmol g^−1^. It seems that a higher charge amount in pyridinic nitrogen (0.33 q) vs. graphitic (0.31 q) (As shown in Table 2), leads to increased adsorption. Pyridinic nitrogen is usually formed in relatively lower temperatures. However, it can also be obtained by conversion of pyridonic-pyrrolic groups at moderate temperatures. On the other hand, at higher temperatures, both pyrrolic and pyridinic nitrogen can change into graphitic types^13,67^. In comparison with graphitic nitrogen, the pyridinic type has higher binding energy. The significant charge transfer from pyridinic nitrogen to CO_2_ leads to a reduction of the CO_2_ bond angle. The highest occupied and the lowest unoccupied molecular orbital energy levels of CO_2_ as well as the slight peak shift in pyridine’s level assist the adsorption process through charge transfer^68^.

### Effect of surface chemistry on CO_2_ capture

**Figure 6:**
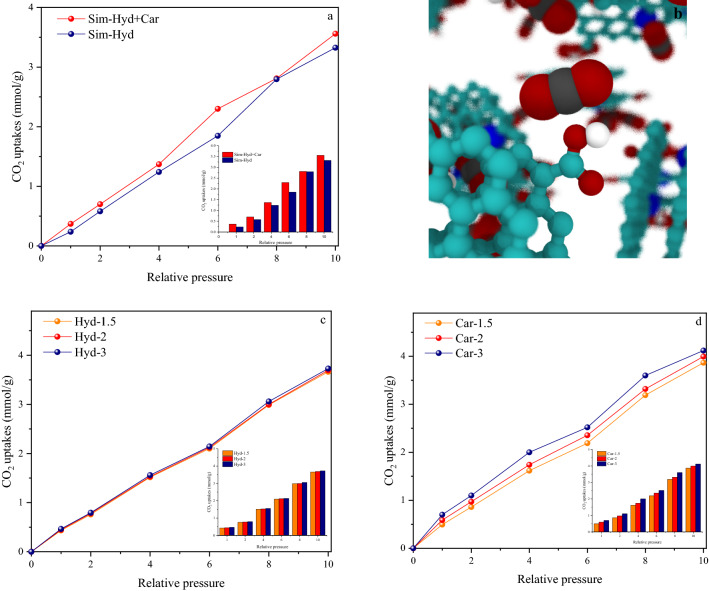
(**a**) Comparison of isotherms simulation at 298 K under pressure range of 0–10 bar within the presence of carboxylic group and without it; (**b**) Snapshot of CO_2_ capture in typical functional group (carboxylic group) present in simulated carbonaceous materials; CO_2_ molecule (Red–black); (**c**, **d**) The amount of CO_2_ capture in different proportions of hydroxyl and carboxyl groups at 298 K under pressure range of 0–10 bar.

The surface of porous carbon, which affects the CO_2_ capturing behavior, can be significantly influenced by oxygen-containing functional groups. The surface chemistry of porous carbon is determined by the structures’ acidity, electronegativity, and hydroxyl groups^69^. To better investigate the role of the carboxylic groups on the enhancement of CO_2_ uptake by MD simulation, these functional groups were added to the initial simulation structure, which contained graphitic nitrogen and hydroxyl groups. As shown in Fig. 6a, the adsorption amount in both the initial structure and the structure that contained carboxyl groups was compared at 298 K and under pressures up to 10 bar. Figure 6b depicts a schematic illustration of the simulated structure containing carboxylic groups as well as CO_2_ adsorption on this structure. The adsorption results indicated that by adding carboxylic groups (The ratio of the carboxyl groups to the hydroxyls is 1:1) to the surface of the simulated structure, the adsorption capacity increases under all measured pressures (10% growth on average) and its maximal value rises from 3.39 to 3.6 mmol g^−1^ at the same conditions. It seems that the oxygenated functionalities such as carboxylic acid groups add a negative charge to the surface and change the electronegativity of the surface^70^. Furthermore, increasing the carboxylic group on the surface improves the surface polarity of porous carbons, which leads to the enhancement of CO_2_ adsorption capacity with a quadrupole moment^71,72^. The carboxyl groups in the framework have the propensity to acquire electrons from their neighboring carbon atoms. Thus they provide Lewis bases reaction and improve bonding interactions with CO_2_ molecules^73^. In other words, the presence of abundant oxygen and the derived carboxylic acids on the simulated structure lead to higher electrostatic interactions owing to their better electron-accepting/donating during the simulation process. This results in the enhancement of CO_2_ capture^74^. Consequently, the simulation findings are consistent with previous research^75^. A snapshot of CO_2_ capture in this condition (Fig. 6b) shows that the CO_2_ molecules tend to be adsorbed near the oxygenated groups (hydroxyl and carboxyl). To better understand the role of functional groups, the values of these groups were increased individually by a certain ratio (The ratio of the hydroxyls groups to the carboxyl are 1.5:1, 2:1, and 3:1 and contrary). It is clear that carboxyl groups play a greater role in CO_2_ uptake than hydroxyl groups as shown in Fig. 6c,d.

### Simulation box-time

**Figure 7:**
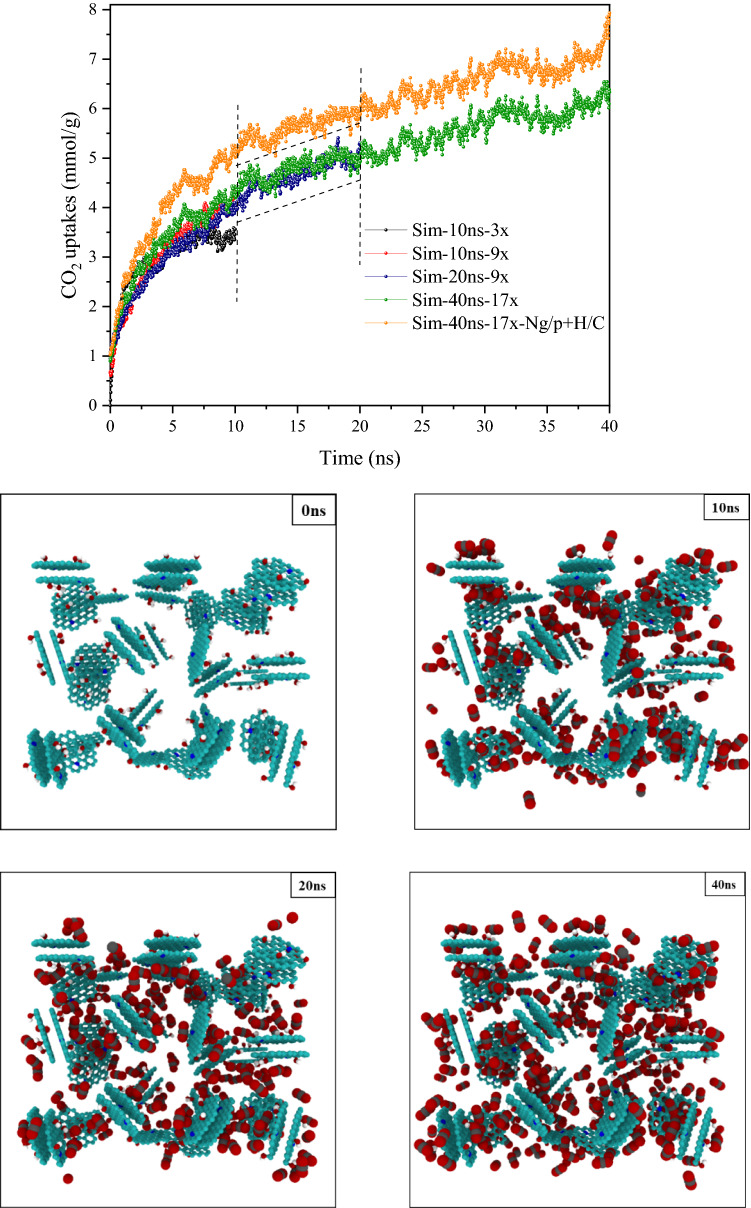
Comparison of the adsorptive capability (at 298 K under 10 bar) in various simulation boxes at 10–40 ns for initial structure and optimal structure.

To evaluate the adsorption results and match them more accurately with the experimental values, the simulation box was modeled in different dimensions vs. the synthesized porous structure and the simulation time was also increased. As shown in Fig. 7, the amount of CO_2_ uptake in the initial simulation box with a size ratio of 1: 3 (The size of the porous structure to the simulation box) was compared with boxes of larger dimensions at 298 K and under 10 bar. Two new simulation boxes with size ratios of 1:9 and 1:17 were generated in this section (Fig. S3). When we used larger boxes we also raised the simulation times. While using the first box, we increased the simulation time to 10 and 20 ns. For the second box, we set the simulation time at 40 ns. Increasing the box’s size up to 1:9 showed major differences in adsorptive properties. It was clear that after the mentioned ratio, increasing the size did not affect the growth of the adsorption and the time (40 ns vs. 20 ns) was the factor that led to the enhancement of adsorption capacity. For the initial box and the initial simulation time (1:3, 2 ns), the adsorption capacity was calculated to be 3.6 mmol g^−1^, while this value reached up to 6.8 mmol g^−1^ for the box size and simulation time of 1:17 and 40 ns, respectively. Given that the final structure contained carboxyl and pyridinic nitrogen, in the simulated structure, we placed four main elements, graphitic-pyridinic nitrogen, and carboxylic-hydroxyl groups, and named it the optimal structure. The optimal simulated structure had an adsorption of 7.95 mmol g^−1^ in the largest box and the longest simulation time (40 ns). The results clearly show that the presence of these four main components in the structure has a much more effective role in gas adsorption than the presence of each of these components separately. Nevertheless, using the larger boxes significantly increased the computational time. It is also clear that according to the results of Table 3, the addition of carboxyl group and pyridinic nitrogen does not have much effect on increasing the specific surface area, which indicates that these parameters do not play a significant role in increasing porosity^76,77^. The density of CO_2_ is determined by using the Van der Waals equation and is the same in all boxes. Because the system is isolated and the number of gas molecules in the initial box stays constant, the total number of molecules in the box is considerably reduced by adsorbing these molecules on the surface of the porous carbon. The density of CO_2_ steadily reduces until the adsorption process ceases. As the size of the box and the quantity of gas molecules rises, the overall density of CO_2_ would have a lower decrease during the adsorption process lowers. As a result, it appears that the adsorptive capability has increased.

**Figure 8:**
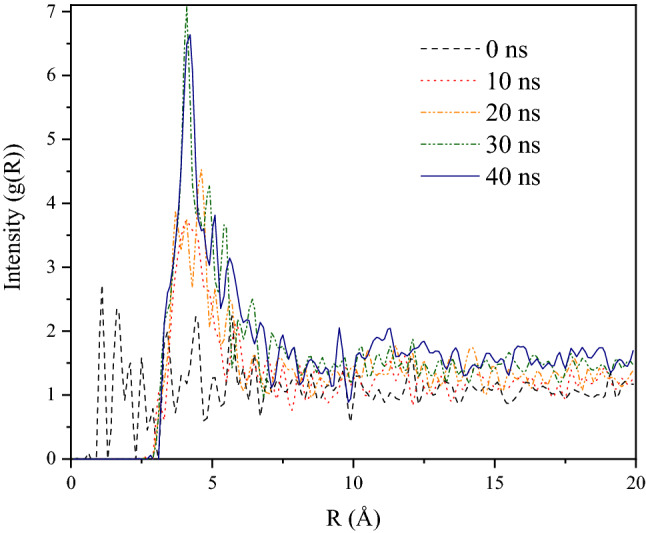
Radial distribution function analyses for CO_2_ (C–C RDF) at different simulation times (at 298 K under 10 bar).

Figure 8 has shown the radial distribution functions (RDFs) for CO_2_ (C–C RDF) at different simulation times under 10 bar, providing a better understanding of the quality of the adsorption process:

6 $$g\left( r \right) = n\left( r \right)\left( {{\rm P}*4\pi *4 \Delta r} \right)$$

where g(r) is the RDF, n(r) is the average number of atoms with width Δr at distance r and Ƥ is the mean atom density. This function is defined as the possibility of finding CO_2_ molecules at r distances from the porous carbon surface in comparison to the probability expected for a completely random distribution at the same density of gas molecules. This figure clearly shows noticeable peaks for the CO_2_ RDFs at distances close to 3.5 Å, indicating single layer adsorption. The second adsorption layer is obscured by the highly irregular and restricted pores.

### Adsorption kinetics

**Figure 9:**
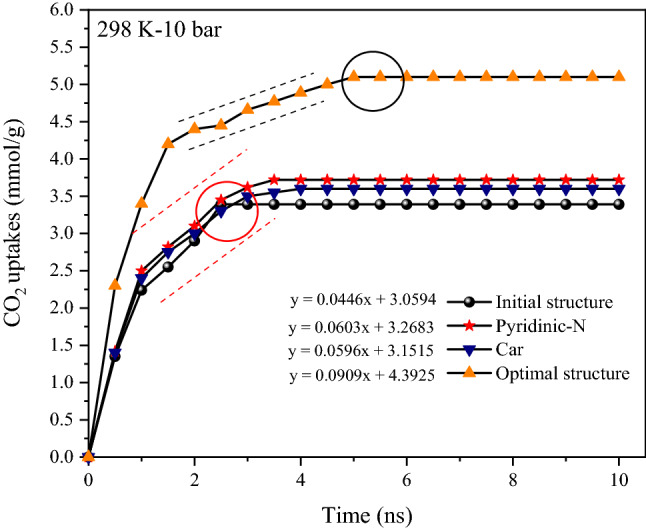
Comparison of gas adsorption kinetics in different conditions (at 298 K under 10 bar). The circles (Black-Red) drawn in the diagram show the starting point of saturation.

All chemistry surface factors affecting the rate of gas sorption may play an effective role in adsorption kinetics. To make studying adsorption kinetics easier, a saturation point is determined, and adsorption fluctuations (± 0.1 mmol g^−1^) are disregarded until the same adsorption rate is considered. The findings are verified up to 10 ns for quicker computation. In addition, the slope type is determined in a linear model. The adsorbed molecules raise sharply at starting of sorption and then becomes nearly flat (Saturation point) as the time increases^78^. The filling of pores and the initiation of adsorption of a layer on the surface of porous carbon are the causes of this occurrence. Collisions increase as the other molecules move closer to the surface, and there is almost no sorption on the surface. As a result, before reaching this phase, we should compare the adsorption rate. The kinetic may be looked at from two different aspects. Because the optimum structure's adsorption capacity is larger than that of other structures, the point of adsorption saturation comes later. This illustrates the important role that all participants have played (Simultaneous effect of carboxyl-hydroxyl groups and graphite-pyridinic nitrogen). Furthermore, the optimal sample's graph has a larger slope than the other samples, and it has adsorbed more gas at a constant time, confirming the structure's faster adsorption kinetics. The presence of pyridinic nitrogen, carboxyl, and hydroxyl groups, respectively, has a stronger effect on the kinetics of the optimum structure (Fig. 9)^79^.

### Prediction of adsorption at very high-low temperatures

**Figure 10:**
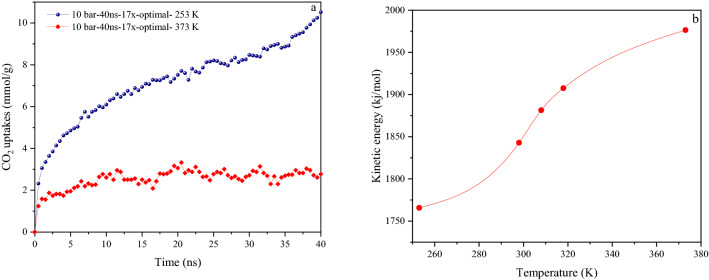
(**a**) CO_2_ adsorption prediction at 253 K and 373 K under 10 bar; (**b**) The kinetic energy of CO_2_ molecules in the range of 253–373 K temperatures.

The amount of CO_2_ sorption at 373 K and 253 K and under 10 bar was predicted to evaluate the type of velocity and motion of the molecules, as shown in Fig. 10a. The box for the test measurement was selected from type Sim-40ns-17x-optimal structure to show a relatively more accurate CO_2_ capture capacity. For a better review, the kinetic energy graphs related to these two temperatures were compared with various temperatures (see Fig. 10b). Cold temperatures are advantageous for adsorption. Thus the adsorption capacity is strongly enhanced below the room temperature. It can be related to gas diffusion and strong interaction between sorbate-sorbent. A sharp drop in temperature indicates that the molecules are moving slowly and as a result, the collision between them greatly reduces. Therefore, the desorption at the simulation surface reduces significantly and the molecules can hardly disperse from the surface, so the adsorption amount reaches up to 10.52 mmol g^−1^. Conversely, at 373 K, the rapid motion of the molecules leads them to have severe collisions with each other that result in a sharp decrease in the adsorption (2.3 mmol g^−1^). The increase in temperature seemed to cause the molecules' motion to change from symmetric to asymmetric^80–82^. To confirm this, the kinetic energy of the atoms (ke/atom) at different temperatures was calculated using the Eq. (6):

7 $$\mathop \sum \limits_{i = 1}^{n} E_{ave} = \frac{1}{2}mV^{2} \times 96.487$$

where m (g) is the mass and V (m/s) is the velocity of each atom. As shown in Fig. 10b, by increasing the temperature up to 373 K, the kinetic energy rises from 1765 to 1976 kJ mol^−1^. The temperature is associated with the kinetic energy and it depends only on the velocity^83^.

### Comparison of isosteric heat of adsorption

**Figure 11:**
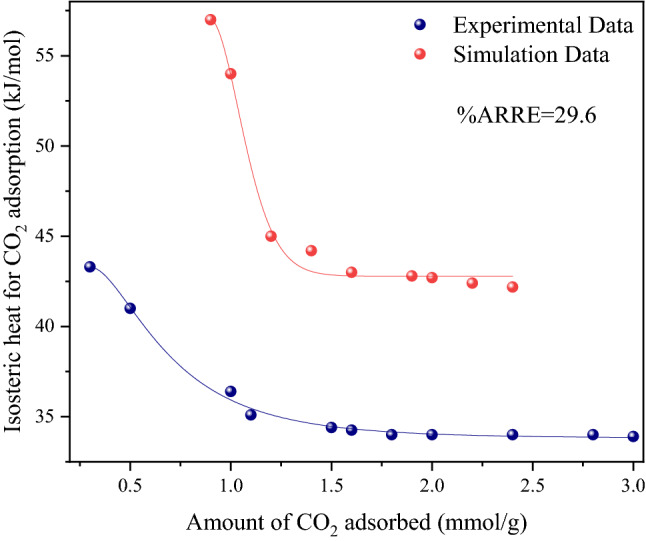
Experimental and simulation CO_2_ isosteric heat of adsorption at three different temperatures of 298, 308, and 318 K.

To gain a better understanding of the adsorption process, the isosteric heat of adsorption, which is a measure of the average binding energy of an adsorbate gas molecule and the surface of the sorbent at a specific surface coverage, was computed at a constant amount of the adsorbed sorbate. By having the isotherm data and using the Clausius–Clapeyron equation, the isosteric heat of adsorption (Qst) was calculated at various temperatures (298, 308, and 318 K). The isosteric heat of CO_2_ adsorption for both situations IS depicted in Fig. 11. After graphing lnP_CO2_ versus 1/T at a set defined adsorbed quantity of CO_2_, the slopes of the straight lines may be used to estimate the Qst value.

8 $$\frac{{ - Q_{st} }}{R} = \left( {\frac{\partial lnp}{{\partial T^{ - 1} }}} \right)_{n}$$

The calculated value of Q_st_ (< 45 kJ mol^−1^) for C-700 sorbent is less than 80 (kJ mol^−1^), indicating that CO_2_ adsorption is physical and similar to results published in the literature on other CO_2_ adsorption cases using ACs^1,84–86^. In simulation results, the Q_st_ values stay in the range of 43.7–57.2 kJ mol^−1^, which indicates higher values than the experimental data and confirms that the adsorption heat was much lower than the energy for chemisorption (> 80 kJ/mol) and covalent bonding. This shows tolerable agreement for accurately capturing the energies of adsorption in the simulation results. For both conditions, as the surface covering and CO_2_ loading increased, the isosteric heat values dropped rapidly and became stable at higher CO_2_ loadings. This variation in Q_st_ can be attributed to a surface that is energetically heterogeneous for CO_2_ adsorption. The greater values of Q_st_ at the initial stage of the adsorption may be attributed to the adsorption of CO_2_ on strong binding sites and the filling of ultrafine micropores. On the other hand, as the surface coverage increases, the values of Q_st_ decrease. This can be explained by weaker interactions between the confined CO_2_ in bigger pores and the surface^53,73,87–89^. The initial simulated Q_st_ value was much greater than the experimental values. The average absolute value of relative error (AARE %) was calculated by Eq. (8):

9 $$AARE\% = \frac{1}{n}\mathop \sum \limits_{i = 1}^{n} \left| {\frac{{X_{exp} - X_{sim} }}{{X_{exp} }}} \right|$$

The simulation results showed slightly more oxygen and nitrogen content and also a much higher amount of hydroxyl (–OH) functional group vs. the experimental results (C-700). Dispersion and electrostatic forces are induced by the interaction between the localized dipoles of the hydroxyl groups and the CO_2_ quadrupole moment, enhancing the Q_st_ value of CO_2_ adsorption. It means that the functional group (hydroxyl) can increase the heat of CO_2_ molecules' initial adsorption on sorbents^74,90–92^.

### Assessment of simulation error

For verification of the simulation results, AARE% was used to determine the accuracy of the simulation in each step, as shown in Table 4. 17x-40ns has the lowest error rate and the highest compliance with the experimental adsorption results (AARE% of 28%). However, the final structure has the most computational time compared to other cases (2614 min). The difference between the MD and the experimental results is discussed in detail and it can be attributed to some factors such as potential deficiencies, different distributions of porosities vs. the experimental specimen, electrostatic charge distribution, absence of some functional groups on the generated surface, and the simulation box’s size^93–95^. Besides, because CO_2_ is a linear molecule, its orientation plays a key role in adsorption onto surface defects, and hence, energetically beneficial sites for the adsorption of a monatomic adsorbate (e.g. the oxygen site in CO_2_) may be unavailable to the polyatomic CO_2_ molecule^96,97^.

**Table 4:** Comparison of various structure properties in simulation cases.

Simulation case	CO_2_ capture (mmol g^−1^)	Computational time (min)	AARE (%)
Initial box (3x-10 s)	3.39	199	64
Initial box + Car (3x-10 ns)	3.6	198	62
N Graphitic + Pyridinic	4.3	198	55
9x-10 ns (Initial)	4.35	220	54
9x-20 ns (Initial)	5.4	892	43
17x-40 ns (Initial)	6.8	2614	28
17x-40 ns + N Graphitic + Pyridinic + Carboxyl (Optimal)	7.95	2620	16

## Conclusions

The purpose of this study was to investigate the behavior of CO_2_ sorption onto porous carbon under different external and internal circumstances. In summary, we synthesized porous carbon (C-700) with a high specific surface area of 1164 m^2^ g^−1^, a high total pore volume of 0.69 cm^3^ g^−1^, and 1.49% N_2_ content. The MD simulation method, using LAMMPS simulator software was applied in combination with the experiments to study the effect of external conditions including time, temperature, and pressure on CO_2_ adsorption rate. The experimental adsorption results as a function of time demonstrated a sharp initial increase in CO_2_ adsorption, which is caused by an enormous number of empty high-affinity pores on the carbon surface and direct contact with CO_2_ molecules that result in strong gas/solid interactions. As the process continues, the pores are occupied and the adsorption reaches a plateau. The experimental procedures are kinetically consistent with the simulation results. Furthermore, the adsorption was examined at different temperatures (298 K to 318 K) and pressures (2 to 10 bar). The highest adsorption amount on both C-700 and the initial simulated structure was achieved at 298 K and under 10 bar with 9.57 and 3.39 mmol g^−1^, respectively. Another aspect we take into account is the influence of internal adsorption parameters including porous carbon’s surface chemistry and its nitrogen type on CO_2_ adsorption. Simulation data acknowledge the previous research by revealing that the adsorption capacity of the simulated structure increased by replacing the graphitic nitrogen with the pyridinic type. On the other hand, by introducing carboxyl functional groups to the surface of the initial simulated structure, CO_2_ adsorption was raised. Eventually, the effect of simulation time and simulation box size on CO_2_ adsorption were investigated. Increasing the initial box’s size ratio-simulation time (From 1:3 and 10 ns to 1:9 and 40 ns) and simultaneous use of all major components in the structure, resulted in adsorption enhancement (From 3.6 mmol g^−1^ to 7.95 mmol g^−1^) and thus the compliance between the simulation and experiments results increased. The adsorption results show the greater effect of the optimal structure (Simultaneous presence of carboxyl-hydroxyl groups and graphite-pyridinic nitrogens) > Pyridinic nitrogen > Carboxylic groups > Hydroxyl groups on the adsorption kinetics, respectively. As a result, by considering the examined factors including nitrogen-carboxylic functionality, simulation time, and size of the simulation box the average absolute relative error percentage (AARE %) for the simulation process declined to 16%. Therefore, this approach can be used as a relatively appropriate method for estimating the CO_2_ adsorption amount at high-low temperatures and pressures that make the experimental measurements impossible.

## Supplementary Information

Supplementary Information.

## Data Availability

All data generated or analyzed data for experimental part during this study are included in this published article [and its supplementary information file]. The simulation datasets generated and/or analyzed in the current study are available upon reasonable request, due to the large associated data volumes. Moreover, all other data that support the plots within this paper and other findings of this study are available from the corresponding author upon reasonable request. MD simulations were performed using the LAMMPS software package. If you need to find out about the structure of the simulation code, you can contact the following emails: mohammadali.abdol@gmail.com, mobinskh@gmail.com.

## References

[1] Ammendola P, Raganati F, Chirone R (2017). CO_2_ adsorption on a fine activated carbon in a sound assisted fluidized bed: Thermodynamics and kinetics. Chem. Eng. J..

[2] Fu N (2017). Iron nanoclusters as template/activator for the synthesis of nitrogen doped porous carbon and its CO_2_ adsorption application. ACS Appl. Mater. Interfaces.

[3] Hong S-M, Choi SW, Kim SH, Lee KB (2016). Porous carbon based on polyvinylidene fluoride: Enhancement of CO_2_ adsorption by physical activation. Carbon.

[4] Wickramaratne NP, Jaroniec M (2013). Activated carbon spheres for CO_2_ adsorption. ACS Appl. Mater. Interfaces.

[5] Zhang X-Q, Li W-C, Lu A-H (2015). Designed porous carbon materials for efficient CO_2_ adsorption and separation. New Carbon Mater..

[6] Park Y, Kang J-H, Moon D-K, Jo YS, Lee C-H (2021). Parallel and series multi-bed pressure swing adsorption processes for H2 recovery from a lean hydrogen mixture. Chem. Eng. J..

[7] Shang J (2020). Separation of CO_2_ and CH_4_ by pressure swing adsorption using a molecular trapdoor chabazite adsorbent for natural gas purification. Ind. Eng. Chem. Res..

[8] Siriwardane RV, Shen M-S, Fisher EP, Losch J (2005). Adsorption of CO_2_ on zeolites at moderate temperatures. Energy Fuels.

[9] Bello G, Garcıa R, Arriagada R, Sepulveda-Escribano A, Rodrıguez-Reinoso F (2002). Carbon molecular sieves from Eucalyptus globulus charcoal. Microporous Mesoporous Mater..

[10] Epiepang FE, Yang X, Li J, Liu Y, Yang RT (2018). Mixed-cation LiCa-LSX zeolite with minimum lithium for air separation. AIChE J..

[11] Ghaemi, A., Mashhadimoslem, H. & Zohourian Izadpanah, P. NiO and MgO/activated carbon as an efficient CO2 adsorbent: characterization, modeling, and optimization. *Int. J. Environ. Sci. Technol.* 1–20 (2021).

[12] Singh G (2019). Biomass derived porous carbon for CO_2_ capture. Carbon.

[13] Saha D, Kienbaum MJ (2019). Role of oxygen, nitrogen and sulfur functionalities on the surface of nanoporous carbons in CO_2_ adsorption: A critical review. Microporous Mesoporous Mater..

[14] Bahadur J (2015). SANS investigations of CO_2_ adsorption in microporous carbon. Carbon.

[15] Ma X (2020). Underlying mechanism of CO_2_ uptake onto biomass-based porous carbons: Do adsorbents capture CO_2_ chiefly through narrow micropores?. Fuel.

[16] Guo Y (2020). Porous activated carbons derived from waste sugarcane bagasse for CO_2_ adsorption. Chem. Eng. J..

[17] Chen C, Hu W, Sun J, Li W, Song Y (2019). CH_4_ adsorption and diffusion in shale pores from molecular simulation and a model for CH_4_ adsorption in shale matrix. Int. J. Heat Mass Transf..

[18] Bahamon D, Ogungbenro AE, Khaleel M, Abu-Zahra MR, Vega LF (2020). Performance of activated carbons derived from date seeds in CO_2_ swing adsorption determined by combining experimental and molecular simulation data. Ind. Eng. Chem. Res..

[19] Zeng H, Liu Y, Liu H (2018). Adsorption and diffusion of CO_2_ and CH_4_ in covalent organic frameworks: An MC/MD simulation study. Mol. Simul..

[20] Zhou G, Liu C, Huang L (2018). Molecular dynamics simulation of first-adsorbed water layer at titanium dioxide surfaces. J. Chem. Eng. Data.

[21] Yang P-Y, Ju S-P, Huang S-M (2018). Predicted structural and mechanical properties of activated carbon by molecular simulation. Comput. Mater. Sci..

[22] Wang H, Qu Z, Bai J, Qiu Y (2018). Combined grand canonical Monte Carlo and finite volume method simulation method for investigation of direct air capture of low concentration CO_2_ by 5A zeolite adsorbent bed. Int. J. Heat Mass Transf..

[23] Chong L, Sanguinito S, Goodman AL, Myshakin EM (2021). Molecular characterization of carbon dioxide, methane, and water adsorption in micropore space of kerogen matrix. Fuel.

[24] Yu X (2020). Effects of helium adsorption in carbon nanopores on apparent void volumes and excess methane adsorption isotherms. Fuel.

[25] Trinh TT, Vlugt TJ, Hägg M-B, Bedeaux D, Kjelstrup S (2015). Simulation of pore width and pore charge effects on selectivities of CO_2_ vs. H_2_ from a syngas-like mixture in carbon mesopores. Energy Proc..

[26] Trinh TT, Tran K-Q, Bach Q-V, Trinh DQ (2016). A molecular dynamics simulation study on separation selectivity of CO_2_/CH_4_ mixture in mesoporous carbons. Energy Proc..

[27] Kim KC, Jang SS (2020). Molecular simulation study on factors affecting carbon dioxide adsorption on amorphous silica surfaces. J. Phys. Chem. C.

[28] Yang Y, Narayanan Nair AK, Sun S (2021). Sorption and diffusion of methane, carbon dioxide, and their mixture in amorphous polyethylene at high pressures and temperatures. Ind. Eng. Chem. Res..

[29] Mashhadimoslem H, Safarzadeh M, Ghaemi A, Emrooz HBM, Barzegar M (2021). Biomass derived hierarchical porous carbon for high-performance O_2_/N_2_ adsorption; a new green self-activation approach. RSC Adv..

[30] Plimpton S (1995). Fast parallel algorithms for short-range molecular dynamics. J. Comput. Phys..

[31] Humphrey W, Dalke A, Schulten K (1996). VMD: Visual molecular dynamics. J. Mol. Graph..

[32] Wang S (2016). Molecular simulation study of the adsorption and diffusion of a mixture of CO_2_/CH_4_ in activated carbon: Effect of textural properties and surface chemistry. J. Chem. Eng. Data.

[33] Kumar KV, Preuss K, Lu L, Guo ZX, Titirici MM (2015). Effect of nitrogen doping on the CO_2_ adsorption behavior in nanoporous carbon structures: A molecular simulation study. J. Phys. Chem. C.

[34] Martínez L, Andrade R, Birgin EG, Martínez JM (2009). PACKMOL: A package for building initial configurations for molecular dynamics simulations. J. Comput. Chem..

[35] Berthelot D (1898). Sur le mélange des gaz. Compt. Rendus.

[36] Lorentz HA (1881). Ueber die Anwendung des Satzes vom Virial in der kinetischen Theorie der Gase. Ann. Phys..

[37] Vella JR (2019). Fick diffusion coefficients of the gaseous CH_4_–CO_2_ system from molecular dynamics simulations using TraPPE force fields at 101.325, 506.625, 1013.25, 2533.12, and 5066.25 kPa. J. Chem. Eng. Data.

[38] Grossman, J., Ferralis, N., Cohen-Tanugi, D. & Dave, S. H. (Google Patents, 2014).

[39] McDonald NA, Jorgensen WL (1998). Development of an all-atom force field for heterocycles properties of liquid pyrrole, furan, diazoles, and oxazoles. J. Phys. Chem. B.

[40] Hermosilla MF, Albesa A (2020). Monte Carlo simulations of simple gases adsorbed onto graphite and molecular models of activated carbon. Adsorption.

[41] Sun C, Wen B, Bai B (2015). Application of nanoporous graphene membranes in natural gas processing: Molecular simulations of CH_4_/CO_2_, CH_4_/H_2_S and CH_4_/N_2_ separation. Chem. Eng. Sci..

[42] Wu D, Yang Y, Liu J, Zheng Y (2020). Plasma-modified N/O-doped porous carbon for CO_2_ capture: An experimental and theoretical study. Energy Fuels.

[43] Wang X-F (2020). Nitrogen-containing porous carbon fibers prepared from polyimide fibers for CO_2_ capture. Ind. Eng. Chem. Res..

[44] Wang H (2019). Hierarchical porous carbon from the synergistic “pore-on-pore” strategy for efficient capacitive deionization. ACS Sustain. Chem. Eng..

[45] Du Y (2020). Template-free preparation of hierarchical porous carbon nanosheets for Lithium–sulfur battery. Langmuir.

[46] Kueasook R (2020). Green and facile synthesis of hierarchically porous carbon monoliths via surface self-assembly on sugarcane bagasse scaffold: Influence of mesoporosity on efficiency of dye adsorption. Microporous Mesoporous Mater..

[47] Vafaeinia M, Khosrowshahi MS, Mashhadimoslem H, Emrooz HBM, Ghaemi A (2022). Oxygen and nitrogen enriched pectin-derived micro-meso porous carbon for CO_2_ uptake. RSC Adv..

[48] Shen F (2018). Oxygen-rich porous carbon derived from biomass for mercury removal: An experimental and theoretical study. Langmuir.

[49] Zhu X, Huang X, Anwer S, Wang N, Zhang L (2020). Nitrogen-doped porous carbon nanospheres activated under low ZnCl_2_ aqueous system: An electrode for supercapacitor applications. Langmuir.

[50] Florent M, Giannakoudakis DA, Bandosz TJ (2017). Mustard gas surrogate interactions with modified porous carbon fabrics: Effect of oxidative treatment. Langmuir.

[51] Goel C, Bhunia H, Bajpai PK (2016). Novel nitrogen enriched porous carbon adsorbents for CO_2_ capture: Breakthrough adsorption study. J. Environ. Chem. Eng..

[52] Guangzhi Y (2017). Preparation and CO_2_ adsorption properties of porous carbon from camphor leaves by hydrothermal carbonization and sequential potassium hydroxide activation. RSC Adv..

[53] Prats H, Bahamon D, Giménez X, Gamallo P, Sayós R (2017). Computational simulation study of the influence of faujasite Si/Al ratio on CO_2_ capture by temperature swing adsorption. J. CO Util..

[54] Liu H, Dai S, Jiang D-E (2013). Insights into CO_2_/N_2_ separation through nanoporous graphene from molecular dynamics. Nanoscale.

[55] Taheri FS, Ghaemi A, Maleki A, Shahhosseini S (2019). High CO_2_ adsorption on amine-functionalized improved mesoporous silica nanotube as an eco-friendly nanocomposite. Energy Fuels.

[56] Taheri FS, Ghaemi A, Maleki A (2019). High efficiency and eco-friendly TEPA-functionalized adsorbent with enhanced porosity for CO_2_ capture. Energy Fuels.

[57] Fu J, Wu J, Custelcean R, Jiang D-E (2015). Nitrogen-doped porous aromatic frameworks for enhanced CO_2_ adsorption. J. Colloid Interface Sci..

[58] Li W (2016). Molecular dynamics simulations of CO_2_/N_2_ separation through two-dimensional graphene oxide membranes. J. Phys. Chem. C.

[59] Liu J, Fan Y-Z, Zhang K, Zhang L, Su C-Y (2020). Engineering porphyrin metal-organic framework composites as multifunctional platforms for CO_2_ adsorption and activation. J. Am. Chem. Soc..

[60] Bonakala S, Balasubramanian S (2015). Modelling gas adsorption in porous solids: Roles of surface chemistry and pore architecture. J. Chem. Sci..

[61] Wei H (2018). Biomass-derived nitrogen-doped porous carbon with superior capacitive performance and high CO_2_ capture capacity. Electrochim. Acta.

[62] Xu L (2015). Nitrogen-doped porous carbon spheres derived from d-glucose as highly-efficient CO_2_ sorbents. RSC Adv..

[63] Wang J (2013). Highly porous nitrogen-doped polyimine-based carbons with adjustable microstructures for CO_2_ capture. J. Mater. Chem. A.

[64] Hao GP, Li WC, Qian D, Lu AH (2010). Rapid synthesis of nitrogen-doped porous carbon monolith for CO_2_ capture. Adv. Mater..

[65] Zhang Z (2019). Rational design of tailored porous carbon-based materials for CO_2_ capture. J. Mater. Chem. A.

[66] Li L, Ma X, Chen R, Wang C, Lu M (2018). Nitrogen-containing functional groups-facilitated acetone adsorption by ZIF-8-derived porous carbon. Materials.

[67] Mashhadimoslem H (2021). Development of predictive models for activated carbon synthesis from different biomass for CO_2_ adsorption using artificial neural networks. Ind. Eng. Chem. Res..

[68] Ma X (2018). Highly nitrogen-doped porous carbon derived from zeolitic imidazolate framework-8 for CO_2_ capture. Chem. Asian J..

[69] Xing X, Jiang W, Li S, Zhang X, Wang W (2019). Preparation and analysis of straw activated carbon synergetic catalyzed by ZnCl_2_-H_3_PO_4_ through hydrothermal carbonization combined with ultrasonic assisted immersion pyrolysis. Waste Manag..

[70] Singh G (2018). A combined strategy of acid-assisted polymerization and solid state activation to synthesize functionalized nanoporous activated biocarbons from biomass for CO_2_ capture. Microporous Mesoporous Mater..

[71] Song X, Wang LA, Gong J, Zhan X, Zeng Y (2020). Exploring a new method to study the effects of surface functional groups on adsorption of CO_2_ and CH_4_ on activated carbons. Langmuir.

[72] Singh G (2020). Emerging trends in porous materials for CO_2_ capture and conversion. Chem. Soc. Rev..

[73] Balou S, Babak SE, Priye A (2020). Synergistic effect of nitrogen doping and ultra-microporosity on the performance of biomass and microalgae-derived activated carbons for CO_2_ capture. ACS Appl. Mater. Interfaces.

[74] Ma X (2019). Experimental and theoretical demonstration of the relative effects of O-doping and N-doping in porous carbons for CO_2_ capture. Appl. Surf. Sci..

[75] Wang Q (2015). Influence of CO_2_ exposure on high-pressure methane and CO_2_ adsorption on various rank coals: implications for CO_2_ sequestration in coal seams. Energy Fuels.

[76] Abbott LJ, Colina CM (2011). Atomistic structure generation and gas adsorption simulations of microporous polymer networks. Macromolecules.

[77] Wang Q, Huang L (2019). Molecular insight into competitive adsorption of methane and carbon dioxide in montmorillonite: Effect of clay structure and water content. Fuel.

[78] Jribi S (2017). Equilibrium and kinetics of CO_2_ adsorption onto activated carbon. Int. J. Heat Mass Transf..

[79] Qin C (2021). Investigation of adsorption kinetics of CH_4_ and CO_2_ on shale exposure to supercritical CO_2_. Energy.

[80] Elfving J, Bajamundi C, Kauppinen J, Sainio T (2017). Modelling of equilibrium working capacity of PSA, TSA and TVSA processes for CO_2_ adsorption under direct air capture conditions. J. CO2 Util..

[81] Samios S, Stubos A, Papadopoulos G, Kanellopoulos N, Rigas F (2000). The structure of adsorbed CO_2_ in slitlike micropores at low and high temperature and the resulting micropore size distribution based on GCMC simulations. J. Colloid Interface Sci..

[82] Haija MA, Romanyshyn Y, Uhl A, Kuhlenbeck H, Freund H-J (2017). Carbon dioxide adsorption on V_2_O_3_ (0001). Top. Catal..

[83] Hamilton BW, Kroonblawd MP, Islam MM, Strachan A (2019). Sensitivity of the shock initiation threshold of 1,3,5-triamino-2,4,6-trinitrobenzene (TATB) to nuclear quantum effects. J. Phys. Chem. C.

[84] Zhou X, Yi H, Tang X, Deng H, Liu H (2012). Thermodynamics for the adsorption of SO_2_, NO and CO_2_ from flue gas on activated carbon fiber. Chem. Eng. J..

[85] Singh VK, Kumar EA (2016). Measurement and analysis of adsorption isotherms of CO_2_ on activated carbon. Appl. Therm. Eng..

[86] Manyà JJ, González B, Azuara M, Arner G (2018). Ultra-microporous adsorbents prepared from vine shoots-derived biochar with high CO_2_ uptake and CO_2_/N_2_ selectivity. Chem. Eng. J..

[87] Deng S (2014). Superior CO_2_ adsorption on pine nut shell-derived activated carbons and the effective micropores at different temperatures. Chem. Eng. J..

[88] Li D (2017). Improving low-pressure CO_2_ capture performance of N-doped active carbons by adjusting flow rate of protective gas during alkali activation. Carbon.

[89] Wu D (2012). Revealing the structure–property relationships of metal–organic frameworks for CO_2_ capture from flue gas. Langmuir.

[90] Ma X (2021). Heteroatom-doped porous carbons exhibit superior CO_2_ capture and CO_2_/N_2_ selectivity: Understanding the contribution of functional groups and pore structure. Sep. Purif. Technol..

[91] Yang J, Yan X, Xue T, Liu Y (2016). Enhanced CO_2_ adsorption on Al-MIL-53 by introducing hydroxyl groups into the framework. RSC Adv..

[92] Mashhadimoslem H, Ghaemi A, Behroozi AH, Palacios A (2020). A New simplified calculation model of geometric thermal features of a vertical propane jet fire based on experimental and computational studies. Process. Saf. Environ. Prot..

[93] Yang Y, Narayanan Nair AK, Sun S (2020). Adsorption and diffusion of carbon dioxide, methane, and their mixture in carbon nanotubes in the presence of water. J. Phys. Chem. C.

[94] Wu X, Huang J, Cai W, Jaroniec M (2014). Force field for ZIF-8 flexible frameworks: atomistic simulation of adsorption, diffusion of pure gases as CH_4_, H_2_, CO_2_ and N_2_. RSC Adv..

[95] Morris W (2012). A combined experimental-computational study on the effect of topology on carbon dioxide adsorption in zeolitic imidazolate frameworks. J. Phys. Chem. C.

[96] Tenney C, Lastoskie C (2006). Molecular simulation of carbon dioxide adsorption in chemically and structurally heterogeneous porous carbons. Environ. Prog..

[97] Zeng K, Jiang P, Lun Z, Xu R (2018). Molecular simulation of carbon dioxide and methane adsorption in shale organic nanopores. Energy Fuels.

