# Supplementary material for: The role of surface chemistry on CO2 adsorption in biomass-derived porous carbons by experimental results and molecular dynamics simulations
Source: Sci Rep. 2022 May 26;12:8917. doi: 10.1038/s41598-022-12596-5 (PMC9135713; doi:10.1038/s41598-022-12596-5)
Supplement: Supplementary file 1 — Supplementary Information. [file 41598_2022_12596_MOESM1_ESM.docx]

Supporting Information (SI)

**The Role of Surface Chemistry on CO_2_ adsorption in Biomass-derived Porous carbons by Experimental Results and Molecular Dynamics Simulations**

Mobin Safarzadeh Khosrowshahi ^1^, Mohammad Ali Abdol^1^, Hossein Mashhadimoslem^2^, Elnaz Khakpour^1^, Hosein Banna Motejadded Emrooz^*,1^, Sadegh Sadeghzadeh^*,^**^1^**, Ahad Ghaemi^*,2^.

^1^ *Nanotechnology Department, School of Advanced Technologies, Iran University of Science and Technology (IUST), Narmak, 16846, Tehran, Iran.*

^2^ *School of Chemical, Petroleum and Gas Engineering, Iran University of Science and Technology (IUST),* *Narmak, 16846, Tehran, Iran.*

* Corresponding Authors

* Tel.: +98 21 73225804; +98 21 73225812; +98 21 77240496

Email corresponding author: [Motejadded@iust.ac.ir](mailto:Motejadded@iust.ac.ir) (H.B.M. Emrooz); [sadeghzadeh@iust.ac.ir](mailto:sadeghzadeh@iust.ac.ir) (S. Sadeghzadeh); [aghaemi@iust.ac.ir](mailto:aghaemi@iust.ac.ir) (A. Ghaemi).


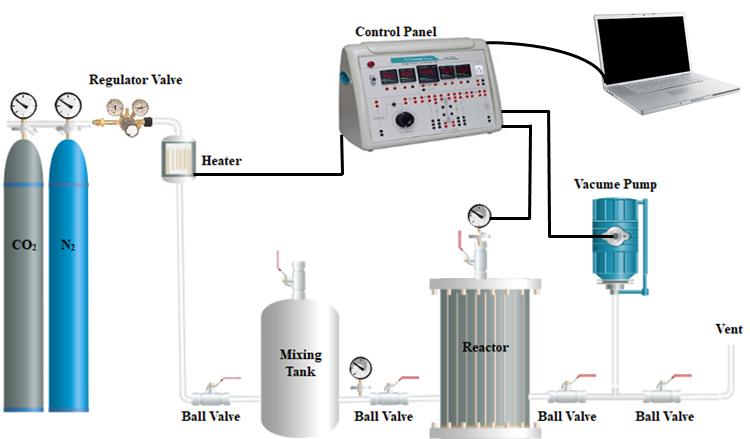


Figure. S.1. Schema of CO_2_ adsorption experimental set-up.

Figure. S.2. Adsorption-desorption simulation curve at 298 K under 10 bar.

**
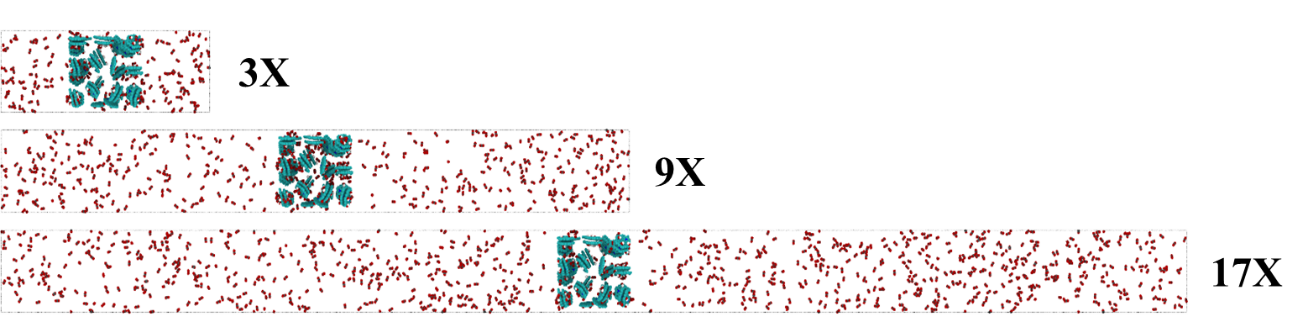
**

Figure. S.3. Simulation boxes with size ratios of 1:3, 1:9 and 1:17.
